# General practitioner referral of older patients to Improving Access to Psychological Therapies (IAPT): an exploratory qualitative study

**DOI:** 10.1192/bjb.2018.10

**Published:** 2018-06

**Authors:** Noel Collins, Laurie Corna

**Affiliations:** 1 Great Southern Mental Health Service, Albany, Australia; 2 Institute of Gerontology, Department of Global Health & Social Medicine, King's College London, UK

## Abstract

**Aims and method:**

To understand general practitioner (GP) reticence to refer older patients to a local Improving Access to Psychological Therapies (IAPT) service providing mostly cognitive–behavioural therapy (CBT)-based interventions. Semi-structured, hour-long interviews were conducted with eight GPs and then analysed by modified grounded theory and thematic analysis.

**Results:**

GP views regarding the treatability of older adults with CBT influenced their willingness to refer to a CBT-based IAPT service. Perceptions of local IAPT assessment processes being distressing and onerous to older patients also motivated referral inaction. GPs expressed a preference to treat depressed older patients themselves (with medication and psychological approaches such as watchful waiting).

**Clinical implications:**

Any strategy to increase referral rates of older adults to CBT-based IAPT services should address local GP concerns regarding assessment processes and the effectiveness of offered treatments.

**Declaration of interest:**

None.

The National Health Service (NHS) England ‘Improved Access to Psychological Therapies’ (IAPT) program was launched in 2009 to improve access to evidence-based talking therapies for people with common psychiatric conditions such as depression. It originally targeted working-age adults but was opened to older adults in 2010, with most interventions being based on cognitive–behavioural therapy (CBT).^1^ Although IAPT is now a service for all ages, only 7% of people in treatment are over 65, despite constituting 20% of the population. This unequal distribution of treatment worsens with age. In particular, there is a miniscule number of people over 90 years old enrolled in IAPT nationally, despite this being the fastest growing demographic group (see https://www.england.nhs.uk/mental-health/adults/iapt/older-people/).^2^ Part of the problem is that people over the age of 65 are rarely referred for psychotherapies.^3^ It is important to understand referral obstacles to IAPT services, as these are now the main providers of talking therapies for adults of all ages.

## Aim

The aim of this study was to understand why GPs did not routinely refer their older patients to a local IAPT provider. This was done by examining the referral decision from the perspective of referrers themselves.

## Method

This was an exploratory, qualitative study. An interpretive approach in the grounded theory tradition was chosen for this research to understand the nuances of the referrer experience and to meanings attached to referral decisions.^4^ Semi-structured, hour-long interviews were conducted with eight GPs practicing in a home county of London. The majority of therapy offered by the local IAPT provider was CBT in nature. The interviewer (N.C.) was a local old-age psychiatrist, previously CBT trained, who was also a member of the Royal College of Psychiatrists Older Adult Faculty work stream aiming to improve access to psychological therapies for older people. An indicative topic guide was provided to all prospective participants in advance of scheduling an interview. As interviews progressed, there was less reliance on interviewer prompts as themes that arose inductively from the data were explored.

Several approaches were employed to mitigate the effects of N.C.'s existing beliefs and assumptions on the qualitative study, including supervision and reflexivity. The latter is an explicit self-awareness of how the interaction between researcher and participant can influence data collection, analysis and subsequent theory development.^5^^–^^7^ The evolution of deductive ideas from previous professional experience and the evolution of inductive theory, as it emerged from the data, were both made explicit. Memo writing was conducted and recorded to provide clear transparency in the analysis.^6^ Care was also taken to delineate worker and researcher roles, and interviews were carried out either out of hours or in non-clinical settings.

### Participants

Purposive sampling of GPs from a variety of backgrounds was carried out to scrutinise deviant examples and increase the scope of subsequent findings.^8^ All eight GPs, who were initially contacted by email, agreed to participate. Half of the GPs were male and half were female. Age and seniority varied between a trainee and commissioner level.

### Ethical considerations

This study was conducted as part of a supervised Master's thesis in Gerontology and was approved by the King's College London Research Ethics Committee prior to the recruitment and interviewing of participants.

### Analysis

Verbatim transcriptions of audio-recorded interviews were made and uploaded to a qualitative software platform (MaxQDA) to assist coding and analysis. Interviews were coded line and by line and analysed using thematic analysis to explore referrer beliefs and their associated meanings, as well as other factors related to referral decisions.^6^ Emergent codes and thematic categories were constantly re-checked regarding their utility in understanding, developing and interpreting emerging themes in the data.^9^ Thematic saturation was observed by the penultimate interview. Several emergent thematic categories, which best captured reluctance to refer to IAPT, were chosen for further examination:

(1) deeming older patients ineligible for CBT;
(2) concern regarding appropriateness of IAPT assessment and treatment;
(3) preferential usage of alternatives to IAPT referral.

These were chosen as the main focus of analysis because these ideas arose inductively from the data and were stable concepts across all transcripts.

## Results

### Theme 1: deeming older people ineligible for CBT

Participants conceptualised anxiety and depression in later life differently to that which occurs in younger adults. This in turn affected practitioner assessments of eligibility for referral, as older adult depression was felt less amenable to CBT for a variety of reasons. These reasons included the beliefs that older adult depression was an inevitable consequence of ageing, loneliness and age-expected losses:

‘Sometimes they have lost children. Work has disappeared. They can't get out and do the things they want to do. Recognising the fact that they are old. And I think people grieve over this almost.’ (GP 4)

‘You're isolated; you're not able to get out. You will get more anxious as you get older.’ (GP 1)

One GP, who had a senior commissioning role, framed his decision not to refer older adults to IAPT using ‘maximising’ health-rationing principles, namely distributing health resources to achieve maximum benefit in a population:^10^

‘I think it's just an unconscious bias, not because I think it becomes less useful as you get older but it's more useful if you are younger. Because I believe that bit about psychological maturity and I think if I took 100 65 year olds and 100 18 year olds, with say anxiety, I believe you would more likely to help more of the 18 year olds rather than the 65s. Because the 65s have probably reached psychological maturity and the 18 year olds haven't. So whilst you might be able to help some of the 65 year olds, you've missed the opportunity of consolidating over a lifetime those patterns of behaviour.’ (GP 8)

GP participants appeared to conflate normal ageing with the development of frailty, sensory or cognitive impairment and the loss of mental agility, which were viewed as obstacles to successful treatment with CBT. This could also explain why non-referral of older patients for CBT was magnified in the over-85 age group:

‘Maybe the older eighty plus person with chronic depression, a bit of dementia, that sort of thing … it's just their life. Is it part of their health, that sort of age? Are we ever likely to make a significant improvement with talking therapy?’ (GP 6)

### Theme 2: concern regarding appropriateness of IAPT assessment and treatment

Some GP participants in this study stated that they did not refer to IAPT due to their concerns that IAPT assessment processes were not suitable for older patients. In particular, telephone assessment and the use of repeated questionnaires were felt to be insensitive and inflexible when assessing older patients, particularly those with comorbid sensory or cognitive deficits. One participant described the abandonment of referral as a ‘rescue’ from the traumatising process of IAPT assessments:

‘I can think of one particular person with early memory loss, you may remember, who was absolutely traumatised by the process … and I said this isn't for you, let's stop all of these appointments and phone calls and all of that … and they've done well, having rescued them from the process they are doing absolutely fine.’ (GP 2)

Other participant concerns regarding IAPT structural processes included inflexible CBT delivery, the poor availability of face-to-face counselling and the skills of IAPT workers in dealing with cognitive impairment or medical comorbidity. The exclusion of nursing home patients was also a particular concern:

‘If I suggested to the homes that these patients be sent to IAPT, they are going to be asking how will we get them there? How will the cognitively, hearing and vision impaired cope? I think that's particularly true with psychologically multi-morbid patients for example: a patient with dementia and anxiety. I suspect you wouldn't refer to IAPT because they wouldn't be able to handle it.’ (GP 8)

A common concern among all GP participants was that IAPT interventions were too short term and superficial to change entrenched maladaptive behaviours in older patients:

‘I think again, most people probably feel as I do in that if you think psychological therapy is going to be helpful – a lot of these people have such strongly held, long term views – that a short course of CBT is not going to do it.’ (GP 4)

All GPs in this sample described feeling isolated from IAPT practitioners. This meant that any concerns regarding the effects of IAPT processes on older patients were never raised with IAPT providers. Additionally, GPs described not receiving any guidance regarding which older patients may benefit from CBT:

‘Obviously that big thing sitting there … where the anxiety that comes in older age, I don't know if that's whom the IAPT service wants to see.’ (GP 1)

### Theme 3: preferential use of alternatives to IAPT referral

When responding to the needs of depressed older adults, GP participants described a number of approaches that are preferentially used instead of referring to IAPT for CBT. These approaches included addressing physical issues and social needs, prescribing medication and GP-led psychological approaches. Prioritising physical issues was viewed as a pragmatic response to the competing demands present in a time-limited consultation with an older patient. It was hoped that by improving physical issues, mood would lift as a result:

‘You also get sidetracked by their UTIs [urinary tract infections] and their arthritis is really bad. I tend to focus on the physical problems as with a lot of the frail people, your assumption is that because they can't get out, they're not mobile, they don't feel well that they're feeling depressed. You therefore focus on those issues – you try to improve those things to improve mood.’ (GP 5)

Addressing social needs, like treating physical issues, was a highly regarded strategy when treating an older adult with depression. Senior GP participants, in particular, had strong convictions in this approach. This was linked to beliefs that older adult depression was often fuelled by social isolation and loneliness, and was therefore more responsive to social interventions rather than psychotherapies:

‘In terms of social interventions that may make a difference, if you were to ask me what differences I've made to people's lives, I can remember a very depressed Finnish lady and I knew close by there was another Finnish lady and I put them in touch, this probably made more difference to her and she didn't come and see me every week after that!’ (GP 7)

Prescribing antidepressants, in contrast to IAPT referral for CBT, was described by some GPs as a reflexive act. Perceived benefits of antidepressants over referral to IAPT included relief of associated insomnia and pain, and taking a tablet being ‘less work’ for patients. Participants also felt that prescribing an antidepressant was more acceptable to older patients and that it satisfied an expectation for a rapid medical response. Some participants also believed that medication was more appropriate than CBT referral when depression had a clear organic cause, had significant somatic symptoms or was associated with medical comorbidity. Concerns regarding side effects were notably absent in all participant accounts. This may relate to the practice of using lower dose antidepressants, described by one GP as ‘gentle pick me ups’:

‘Again it comes down to expectation, if they leave with a prescription in their hand, you've done something. Whereas, if I say to them I'll refer you to a counselling service, it'll take 6–8 weeks before you see somebody, then might have to wait for treatment and in 4 months time, you might be engaged with the service and you might start to feel better … you're not giving them a quick fix.’ (GP 5)

In contrast to referral to IAPT, GPs reported that prescribing made them feel less impotent and helpless:

‘With a depressed elderly, you worry that they are going to wait so long [for CBT]. I must do something in the interim. Doctors hate that hopeless feeling. You know what you need to do, but you can't access that.’ (GP 5)

GPs described using their own psychological skills with older patients with depression rather than referring them to CBT. These approaches included supportive counselling, exploratory brief therapy and problem solving. Like prescribing medication, these strategies were deemed within the GP's control:

‘You learn the older you get that some people just want to acknowledge an issue and they have it within themselves, and having acknowledged and having told someone like their GP about it, that they're able then to go away … and … change their thinking about it.’ (GP 2)

Watchful waiting was frequently used as a tried-and-trusted approach to treating elderly people with depression in primary care. This intervention, centred on regular review but without active pharmacotherapy or psychotherapy, was felt by participants to be a tangible and containing approach compared with referral to IAPT for CBT:

‘I think it's the personal support and contact, the commitment. Because quite often people who I think need a lot of support, I will book the next appointment whilst they are in the room and give them that piece of paper. So again, it's that tangible thing. Even if I don't think they need medication, I will say let's find an appointment. And they will go out and come back.’ (GP 4)

Some participants felt confident in delivering simpler CBT interventions themselves, such as behavioural activation, activity scheduling and challenging negative thinking. The willingness and desire to improve these skills, even within the constraints of brief consultations, was most clearly expressed by a GP registrar:

‘I think the more you learn about CBT, the more you can do very basic interventions yourself with some patients. We've been encouraged to do that and deliver very basic CBT ourselves. In the 10 minute consultation!’ (GP 5)

### Study limitations

The findings presented here must be interpreted in light of the study's limitations. The IAPT service referred to in this study was reported by local GPs to only offer only CBT-based therapies. Participants had strong views regarding the use of CBT in older patients and this clearly influenced willingness to refer to their local service. The findings of this study cannot be generalised to the experiences of other GPs with other IAPT providers. Additionally, the sample comprised GP participants in only one semi-rural locality with little ethnic diversity. This meant that the experiences of more urban-based GPs working with multicultural populations were also absent. The scope of the project precluded a larger sample size, but data saturation was achieved by the seventh interview. Notwithstanding these caveats, this study does provide some insight into the reasons for underutilisation of IAPT services by older adults.

## Discussion

The GP respondents in this study gave a wide range of clear reasons for their reluctance to refer their older patients to a local IAPT provider offering mostly CBT. These include the belief that older adult depression was an inevitable consequence of ageing and therefore more difficult to treat with CBT. This is consistent with previous research showing how depression in older adults can be viewed as a ‘justifiable’ consequence of ageing due to age-expected losses and social changes, and therefore less in need of treatment.^11^^–^^13^ This view may in turn relate to historical comments by Freud, e.g. ‘above the age of 50 the elasticity of mental processes on which treatment depends is, as a rule lacking – old people are no longer educable’, still having an enduring effect on medical practitioners.^3^

In general, GP participants in this study viewed IAPT assessment processes as inflexible, insensitive and potentially traumatising for older adults. This is compatible with existing concerns that CBT can be too protocol driven, measurement focused and inflexible for older patients.^14^^,^^15^ There appeared to be some ‘frail-ist’ views where the older, more frail, depressed individuals in particular were deemed the least likely to benefit from or access CBT and therefore were ineligible for referral. The treatment preferences of GPs in this study are consistent with existing research showing that some GPs prefer simpler and more exploratory counselling rather than a manualised approach like CBT,^16^ and that they believe that pharmacotherapy offers quicker relief to people with depression compared with a talking therapy.^17^

The results of this study point to strategies that could achieve higher referral rates for older patients to IAPT services. Increased co-location of IAPT therapists into primary care would be a good starting point in promoting CBT as an effective treatment for older people, who often have higher rates of adherence and completion of therapy than younger patients.^15^^,^^18^ Some IAPT providers could aim to offer a greater range of other modalities of treatment to older patients and publicise these increased options to referring GPs. IAPT providers could also revise assessment processes, such as the use of simpler rating scales of improvement, to address concerns regarding onerous outcome measurement. The use of tele-health could also be trialled to improve access for those with mobility impairments and those living in residential homes.^19^ IAPT supervisors could also take on an active role in training carers in residential homes to deliver basic CBT techniques to depressed, older and frail adults who are currently disenfranchised by poor access to conventional face-to-face treatment.^20^ It is hoped that this paper helps raise the profile of unequal access to psychological therapies for older adults and that some of the suggested strategies derived from this study's findings can be trialled by some IAPT services to improve the situation.

## About the authors

**Noel Collins** is a consultant psychiatrist at the Greater Southern Mental Health Service, Albany, Australia. **Laurie Corna** is a senior lecturer at the Institute of Gerontology, Department of Global Health & Social Medicine, King's College London, UK.
